# An improved algorithm for inferring mutational parameters from bar-seq evolution experiments

**DOI:** 10.1186/s12864-023-09345-x

**Published:** 2023-05-06

**Authors:** Fangfei Li, Aditya Mahadevan, Gavin Sherlock

**Affiliations:** 1 Department of Genetics, Stanford University, Stanford, California US; 2 Department of Physics, Stanford University, Stanford, California US

**Keywords:** Experimental evolution, Genetic barcoding, Inference

## Abstract

**Background:**

Genetic barcoding provides a high-throughput way to simultaneously track the frequencies of large numbers of competing and evolving microbial lineages. However making inferences about the nature of the evolution that is taking place remains a difficult task.

**Results:**

Here we describe an algorithm for the inference of fitness effects and establishment times of beneficial mutations from barcode sequencing data, which builds upon a Bayesian inference method by enforcing self-consistency between the population mean fitness and the individual effects of mutations within lineages. By testing our inference method on a simulation of 40,000 barcoded lineages evolving in serial batch culture, we find that this new method outperforms its predecessor, identifying more adaptive mutations and more accurately inferring their mutational parameters.

**Conclusion:**

Our new algorithm is particularly suited to inference of mutational parameters when read depth is low. We have made Python code for our serial dilution evolution simulations, as well as both the old and new inference methods, available on GitHub (https://github.com/FangfeiLi05/FitMut2), in the hope that it can find broader use by the microbial evolution community.

**Supplementary Information:**

The online version contains supplementary material available at 10.1186/s12864-023-09345-x.

## Background

Clonal interference is a phenomenon that occurs in large asexual populations, in which multiple beneficial mutations arise contemporaneously and compete with each other without recombining onto the same genetic background [1–5]. Although these mutations may later accumulate onto the same genomes due to a high mutation rate [6], clonal interference remains an important evolutionary force across a wide range of timescales. Experimental evolution in large microbial populations, where the emergence of beneficial mutations is common enough that clonal interference is widespread, has been widely used to explore this regime of adaptation [7–11]. In such experiments, microbes such as fungi, bacteria, or viruses are propagated for hundreds (or thousands) of generations in a controlled experimental system, typically either by serial transfer of batch cultures or continuous culture. Adaptive mutations that emerge during such an experiment can be identified by whole-genome sequencing (WGS) of multiple isolates from the evolved populations [12–15].

However, this approach has limitations. Firstly, it cannot provide information about the occurrence time and fitness effect of mutations. Secondly, it can only identify the subset of adaptive mutations that reaches high frequency, which tends to consist of those which arose earlier or provide a larger fitness benefit. Although the minimum frequency at which the mutation is detectable can be lowered by sequencing more isolates, the high cost of WGS (in comparison to amplicon sequencing) quickly makes this method impractical for identifying low-frequency mutations. An alternative to WGS for multiple isolates at the end of an evolution experiment is to conduct WGS for the whole population at multiple time points during the evolution [11, 16, 17]. With such a time series, one can roughly estimate the occurrence time of some of the mutations. However, this method is more expensive, does not provide the fitness effects of mutations, and fails to identify mutations at a frequency lower than $\sim 1\%$ under typical financial constraints (assuming a single flow cell yields $\sim 10^9$ read pairs, which is sufficient to sequence genomes pooled from $\sim 10$ timepoints to $\sim 100\times$ depth) and due to sequencing and/or PCR errors.

To better explore these low-frequency dynamics, a high-resolution lineage tracking system was developed in *S. cerevisiae*, based on a genetic barcoding platform [18]. This system is capable of detecting thousands of initial adaptive mutations in an originally clonal evolving population, by simultaneously monitoring the relative frequencies of $\sim 5\times 10^5$ lineages, each defined by a unique genetic barcode of 20 nucleotides and consisting of $\sim 100$ cells initially in a typical experiment [18]. With the high-resolution information on lineage frequencies from the barcode counts at multiple time points, one can estimate fitness effects and establishment times of adaptive mutations using a statistical framework based on the theory of branching processes and Bayesian inference (as done in [18]). Here we refer to this algorithm as FitMut1. FitMut1 can detect adaptive mutations at frequencies higher than $\sim 10^{-6}$ from barcode frequencies over time, and can be followed by WGS for clones with different barcodes for further characterization of mutations at the genotypic level. For this step, isolating clones is relatively straightforward since each lineage contains an unique barcode that can be easily recognized by Sanger sequencing [19]. In addition to *S. cerevisiae*, other microbes such as *E. coli* have been studied with similar barcoding approaches [20, 21].

It should be emphasized that not all beneficial mutations are detectable. A minimum requirement for a mutation to be detected is its *establishment* [2, 18]. For a beneficial mutation that occurs initially in a single cell and with fitness effect *s*, there is a substantial probability of going extinct soon after occurring, due to random fluctuations, even though the mutant confers a growth advantage. However, if a mutant gets “lucky enough” (with the probability proportional to *s*) to reach a certain size (proportional to 1/*s*), it will grow exponentially with rate *s* thereafter. In this case, we say that the mutation carried by the mutant has *established*. By extrapolating its exponential growth backward in time until the mutant population crosses the rough boundary between stochastic and deterministic dynamics, we can define an *establishment time* as the time after which the mutant cells effectively grows deterministically. Establishment time roughly reflects the occurrence time of a mutation, up to uncertainty on the order of 1/*s*.

Nevertheless, the exponential growth rate of an adaptive lineage (in which an adaptive mutation has established) cannot be measured directly to yield the fitness effect of the mutation. This is because 1) the mutation must sweep through the entire lineage before dynamics of the lineage reflect those of the mutation, and 2) the lineage trajectory in a well-mixed environment bends over as it competes against the increasing population mean fitness. Therefore, the mean fitness is required for accurately characterizing the dynamics of adaptive lineages and further inferring establishment times and fitness effects of mutations. However, it is difficult to measure the mean fitness directly. In FitMut1, the mean fitness is estimated by monitoring the decrease in frequency of neutral lineages (those without an established mutation) between consecutive sequencing time points. However, FitMut1 fails when the number of available neutral lineages is insufficient, which can happen when the sequencing read depth is low, the bottleneck size (number of cells per barcode at the bottleneck) is small, or the mean fitness increases rapidly.

In this work, we describe an improved algorithm, FitMut2, which iteratively estimates the mean fitness and characterizes the adaptive lineages in an evolving population, without relying on the number of available neutral lineages. This makes FitMut2 less impaired by low sequencing read depth, small bottleneck size, or rapidly increasing mean fitness, and thus more robust and accurate than FitMut1. To assess the performances of both algorithms, we ran FitMut1 and FitMut2 on the same simulated dataset and compared their outputs with the ground truth.

We first introduce FitMut2, which includes a summary of FitMut1 and the modifications that constitute FitMut2 (Methods section). In addition, we also discuss the simulated data that we used to benchmark the performance of FitMut2 and compare it to FitMut1 (Methods section). We then evaluate the performance of FitMut2 on simulated data and compare it with FitMut1 on the same dataset (Results section). Finally, we discuss the limitations of FitMut2 and possible future improvements (Discussion section).

## Methods

### Algorithm overview

FitMut1 models the dynamics of lineage growth with a stochastic branching process, thereby associating with each lineage a probability distribution of the number of reads that map to its barcode, conditional on this read number at a previous time point. In addition to the demographic stochasticity of births and deaths, this distribution considers various sources of noise: cell transfer, DNA extraction, PCR and sequencing, which are represented by a phenomenological parameter $\kappa _k$ for each time point $t_k$ (details in Supplement S4). Our noise model is consistent with a branching process, wherein (conditional on the read number at $t_{k-1}$) the variance in read number at $t_k$ is proportional to the mean read number at $t_k$, with constant of proportionality $2\kappa _k$. In FitMut1, first the mean fitness of the population $\bar{s}(t_k)$ and the noise parameter $\kappa _k$ are estimated for all sequencing time points $t_k$ by monitoring the decline of neutral lineages, which are assumed to constitute the majority of lineages at intermediate read number. Assuming that each lineage contains a mutation with fitness effect *s* and establishment time $\tau$, the posterior likelihood of each lineage trajectory data is calculated, and maximized over *s* and $\tau$ to find the values for which the observed data are most likely. FitMut1 relies on neutral lineages for estimating the mean fitness, which then influences all subsequent steps. Although this works well when the number of neutral lineages is large (e.g. at the beginning of a typical experiment), the number of neutral lineages falls dramatically during the evolution as beneficial mutations increase in frequency in the population, and the efficacy of FitMut1 thus decreases.

Instead of relying on neutral lineages, FitMut2 uses an iterative approach to self-consistently infer the population mean fitness together with *s* and $\tau$ for each putative mutation. This approach does not require a large number of neutral lineages to be present and enforces that the individual mutations and their frequencies are consistent with the inferred population mean fitness. FitMut2 only relies on the number of putatively neutral lineages to estimate the noise parameter $\kappa _k$ at each sequencing time point, and we have found that the inference results are not very sensitive to the value of this parameter. With this self consistent method, FitMut2 identifies more adaptive mutations and obtains the *probability* of a lineage being adaptive conditional on the data. By contrast, FitMut1 provides a ratio of posterior likelihoods, which is not required to be between 0 and 1, and is harder to interpret. The algorithm of FitMut2 proceeds as follows:

1. For each sequenced time point: Initialize the mean fitness to 0 and calculate $\kappa _k$ from the empirical distribution of read numbers assigned to putatively neutral lineages at that time.
2. For each lineage: Use Bayes’ theorem to calculate the probability that the lineage is adaptive given the observed read number trajectory, under a prior distribution over fitness effect *s* and establishment time $\tau$ (the choice of prior is discussed further in the Discussion section). If the probability is greater than 0.5, designate the lineage adaptive, and maximize the posterior likelihood over *s* and $\tau$ to find the most likely fitness effect and establishment time under the assumed prior (see details in details in Supplement S5).
3. Estimate the number of mutant cells over time for each identified adaptive lineage, using the inferred fitness effect and establishment time of the mutation. Update the mean fitness accordingly.
4. Repeat steps 2 and 3 until the estimated mean fitness at each sequencing time point converges to a self-consistent value.

By design, the mean fitness $\bar{s}(t_k)$ at time point $t_k$ must agree with the mean fitness from the adaptive lineages and their frequencies, given by $\sum _i s_i f_{i, k}^\text {mut}$ where $s_i$ is the fitness effect of the mutation of lineage *i* and $f_{i, k}^{\text {mut}}$ is the frequency of mutant cells at time point $t_k$. Although these two ways of estimating the mean fitness are shown to roughly agree in FitMut1 [18], their equality is explicitly enforced here, and it improves the algorithm’s accuracy when the read depth is low or the mean fitness is rapidly increasing.

### Simulation

**Fig. 1:**
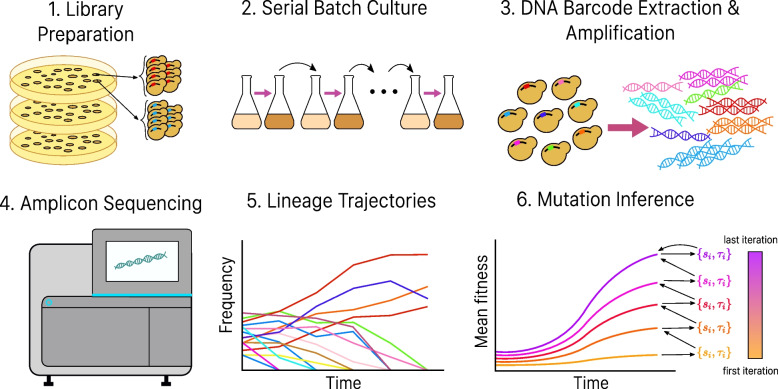
Procedure of a complete barcoded evolution experiment with analysis included. Steps 1 to 4 depict the procedure of a typical bar-seq evolution experiment, which gives rise to a series of lineage trajectories over the course of the experiment (Step 5), with each lineage defined by one barcode. Different colors represent lineages with different barcodes. Step 6 is a schematic of how we use these trajectories to identify adaptive mutations that occurred in the evolution experiment, and self-consistently infer the fitness effects and establishment times of individual mutations together with the mean fitness of the population (Methods section)

Numerical simulation is an effective method to evaluate performance of the algorithm when available experimental data are limited. Here, we evaluated the performance of FitMut2 using a simulated dataset, which allows us to compare the inference result with the ground truth. Our numerical simulations consider the entire process of a barcode-sequencing (bar-seq) evolution experiment of a barcoded cell population using serial batch cultures (Fig. 1). Starting from a single cell per barcode, lineages first go through 16 generations of pregrowth without competition, during which mutations can and do occur. This step simulates the cell growth on agar plates after the barcode transformation but before the evolution experiment begins. Each cell into which a barcode was successfully transformed can grow into a colony on an agar plate, with all cells in the colony containing the same barcode. The pregrowth phase includes two processes which are inevitable in the experimental process of building a barcode library: 1) the growth noise on agar plates, which generates a non-uniform distribution of lineage sizes and 2) the occurrence of mutations before the evolution experiment commences. Both of these features can significantly influence the evolutionary dynamics. After being scraped from agar plates, cells of the colonies are pooled together and grown up overnight before being sampled and inoculated into the medium. In the simulation, we ignore this process, because it includes very few generations of growth.

To initialize the evolution experiment with a barcode library consisting of *L* unique barcodes, 100*L* cells are sampled from the population after the pregrowth. This yields a mean of 100 cells per barcode, which roughly corresponds to parameters commonly used in an experiment. The barcoded population is then evolved through pooled growth by serial batch culture. Each growth cycle consists of *g* generations of stochastic doubling, after which a fraction $1/2^{g}$ of the cells from the end of the growth cycle is sampled and transferred to another fresh culture. This process of growth and dilution, with *B* cells transferred at each bottleneck, can be thought of as constant-population process with an *effective population size* given by *gB* and a per-generation offspring number variance $2c \approx 2$ (more details discussed in Supplement S3). This effective description is very useful for quantitatively matching theory to experiment, and is essential for the functioning of both FitMut2 and FitMut1. Hereafter, we use the term *effective lineage size* to refer to the lineage or population size that would be necessary to obtain the same statistics of lineage fluctuations if the total number of cells was constant in time rather than growing by a factor of $2^{g}$ every cycle.

Although our simulations can keep track of an arbitrary number of mutations per cell, we have not pursued the inference of these later mutational effects in the current work. Instead we make the simplifying hypothesis that at most one beneficial mutation occurs per cell. In light of evidence suggesting that the distribution of fitness effects (DFE) of the second mutation in a cell is different from that of the first mutation, due to epistasic or physiological constraints [22], this hypothesis allows us to focus on *initial* adaptive mutations. Although each simulated individual can obtain at most one beneficial mutation, only $\sim 3\%$ of lineages contain more than one established mutation in our simulations; when this occurs we record the “true” fitness of the lineage as the *s* of the mutation that generates the maximum number of mutant cells by the end of the evolution. A mutation that occurred with fitness effect *s* is counted as established if, at any time during the evolution, the mutant’s instantaneous frequency reaches $2c/(N(s-\bar{s}(t)))$. We have found that typically, on the order of 20 high fitness mutations are sufficient to account for the majority of the mean fitness increase over the simulation. However we are able to identify many more mutations than these, though they do not contribute substantially to the mean fitness.

At each cell-transfer time point over the course of the experiment, 500*L* cells are sampled from the saturated population to simulate the process of genome DNA extraction, and go through 25 rounds of stochastic doubling, to simulate PCR with 25 cycles (a larger number of cycles will not have a significantly larger effect on the PCR noise, since only the initial doublings contribute to the stochasticity). Then an extra sampling of size *rL* is performed after PCR to simulate the noise introduced by sequencing, with *r* being the average sequencing read number per lineage per time point.

The entire process generates a lineage trajectory over time for each barcode. The simulation includes five potential sources of noise: cell growth, sampling during cell transfers, DNA extraction, PCR, and sequencing. Each step is modeled by a layer of Poisson noise (including for each generation of cell growth and each cycle of PCR). To test the performance of FitMut2, we ran simulations with four different underlying DFEs denoted by $\mu (s)$, where $\mu (s)ds$ is the rate of mutations with fitness effect in the interval $(s,s+ds)$ (details of the DFEs we simulated are in Supplement S8). The total beneficial mutation rate is given by $U_b = \int _0^\infty \mu (s)ds$. For each simulation, a population of $4\times 10^4$ single cells undergoes 16 generations of pregrowth before all these lineages are pooled and grown by serial batch culture for $T = 112$ generations, with ${g} = 8$. For each of four DFEs, sequencing is simulated with four different average read numbers per lineage $r = 10, 20, 50, 100$.

Inference is performed with the same prior distribution for all conditions: $p(s, \tau ) =n_{i, 0} \tilde{\mu }(s) s/c$. Note that $\int p(s,\tau ) ds d\tau$ is approximately the number of established mutations per lineage. The factor of *s*/*c* arises from establishment probability $\sim s/c$ in the branching process model, and $n_{i, 0}$ is the effective size of lineage *i* at $t_0$. $\tilde{\mu }(s)$ is the prior we take for the DFE, which is $\tilde{\mu }(s) = U_b \lambda ^{-1} e^{-s/\lambda }$ with $\lambda = 0.1$ and $U_b = 10^{-5}$ throughout this paper.

**Fig. 2:**
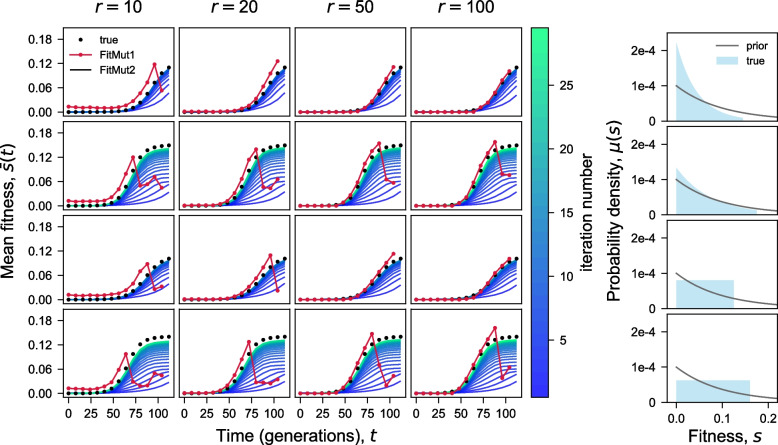
Iterative inference of the mean fitness. Comparison of the true mean fitness $\bar{s}(t)$ with the mean fitness inferred by both FitMut1 and FitMut2, for different sequencing depths (columns) and $\mu (s)$ (rows). Each row in the $4\times 4$ array corresponds to one simulation of the evolution, with the columns differing by the average simulated sequencing depth per time point per lineage. The 5th column shows the DFE $\mu (s)$ used in each simulation condition, and the prior $\tilde{\mu }(s)$ used for both FitMut2 and FitMut1 across all simulated DFEs

Figure S1 shows trajectories of all lineages in one of our simulations with an exponential DFE and $r=100$, corresponding to the 1st row and 4th column in Fig. 2.

## Results

### Fitness per generation vs. fitness per cycle

Before presenting the results of running FitMut2 on our simulated dataset, we discuss an important aspect of interpretation that should be kept in mind whenever one is analyzing data from serial batch culture. How should we interpret the parameters output by our inference algorithm? Specifically, what is the meaning of the fitness increment *s* obtained by a particular lineage in a biological sense? Previous work has explored the importance of variable growth conditions *over a single cycle of batch culture* in creating a much more complex environment than meets the eye [23]. In particular, selection pressure varies over a single cycle of batch culture as the environment  shifts dramatically according to the metabolic processes being performed. Within a single cycle of batch culture spanning *g* generations, the relevant quantity for the evolutionary dynamics is not *fitness per generation*, but rather *fitness per cycle*. In fact, many adaptive mutations may have time-varying fitness effects over a single cycle. In *S. cerevisiae*, which undergoes multiple metabolic shifts over a single growth cycle, a beneficial mutation can be neutral during the first seven generations (fermentation), but have a large fitness advantage during the last generation of the cycle (respiration). Therefore it is misleading to interpret results in terms of a fitness per generation: we cannot define a singular notion of “fitness” without accounting for the interaction between organism and environment. One should avoid claiming more granularity than one’s highest temporal resolution, which here is the length of a single batch culture cycle. Since the theory on which FitMut2 is based considers a simpler effective model without time-varying fitness, fitness effect per generation is used in the branching process model we use. However, the trouble arises if the theory is taken too seriously in interpreting experimental results. Although we report results in terms of fitness per generation in our inference algorithm for both the population mean $\bar{s}(t)$ and for adaptive mutation *s*, we emphasize that in real experimental conditions there is little reason to believe that these values correspond to anything other than an *average* quantity across a cycle that depends on experimental setup and conditions.

### FitMut2 robustly estimates mean fitness

FitMut2 and FitMut1 differ essentially in how they infer the population mean fitness — this then leads to differences in the inference of mutational parameters. Figure 2 shows the mean fitness trajectories inferred by both FitMut2 and FitMut1. The number of iterations required to converge upon a self-consistent mean fitness is larger for simulations with a wider DFE, but rarely exceeds 40, and convergence appears monotonic. FitMut1 estimates the mean fitness accurately when sequencing read depth is high (i.e. $r=100$), and the mean fitness increases slowly (all time points for DFEs with small variance, or early time points for DFEs with large variance). However, for low sequencing read depth ($r=10$ or 20), or as the mean fitness increases rapidly (later time points for DFEs with large variance), FitMut1 begins to perform poorly.

### FitMut2 accurately estimates mutational parameters

**Fig. 3:**
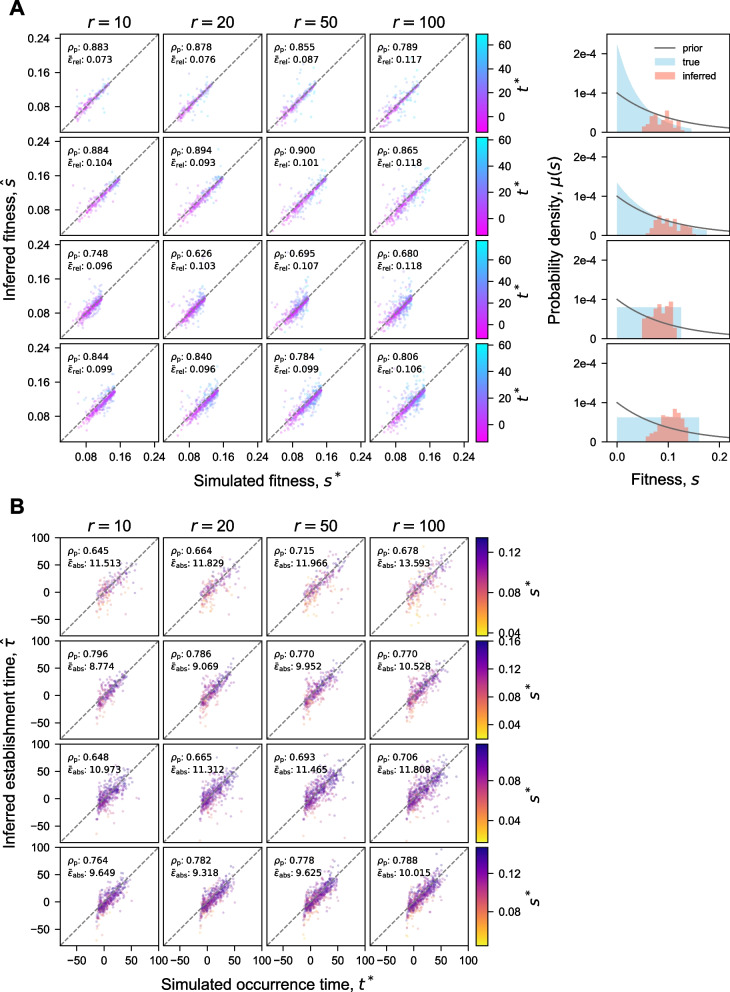
Inference accuracy of **A** fitness effects and **B** establishment times (FitMut2). Comparison of inferred *s* and $\tau$ with simulation. Each panel in the $4\times 4$ array corresponds to one simulation (Methods section). Each point is an adaptive mutation that established in the simulation and was identified by FitMut2. Points in (**A**) are colored by their true occurrence time $t^*$, while points in (**B**) are colored by their true fitness $s^*$. Negative $t^*$ indicates adaptive mutations that occurred during pregrowth. $\epsilon _{\text {rel}}$ in (**A**) is defined as $\frac{|s^* - \hat{s}|}{s^*}$ and $\epsilon _{\text {abs}}$ in (**B**) is defined as $|t^* - \hat{\tau }|$. $\rho _p$ is the Pearson correlation coefficient. The 5th column in (**A**) shows the comparison between $\mu (s)$ and the inferred DFE (estimated as in Supplement S8). In Fig. S2 we show the same data for FitMut1

**Fig. 4:**
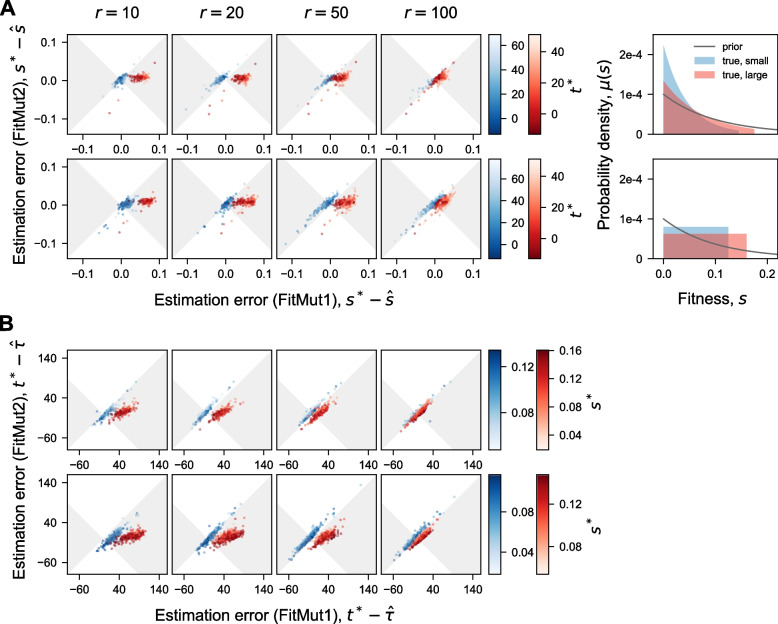
Estimation error of the fitness effects and the establishment times. Comparison of the estimation error, measured between simulation and inference, for FitMut1 and FitMut2. **A** shows fitness effects and **B** shows establishment times. Each column corresponds to an average number of reads per lineage *r*. Different rows correspond to different classes DFEs: exponential and uniform. Each panel includes the inference error of two simulations (Methods section) from the same family of $\mu (s)$ with different variances (blue for smaller variance, red for larger variance). Each dot in the scatter plot represents an adaptive mutation that established in the simulation and was identified by both FitMut2 and FitMut1. Dots falling within the gray region indicate the adaptive mutations that were more accurately inferred with FitMut2 than with FitMut1. Blue and red dots are colored by their occurrence time $t^*$ for (**A**), and by their true fitness $s^*$ for (**B**). $\mu (s)$ is plotted in the 5th column (blue for small variance, red for large variance)

**Fig. 5:**
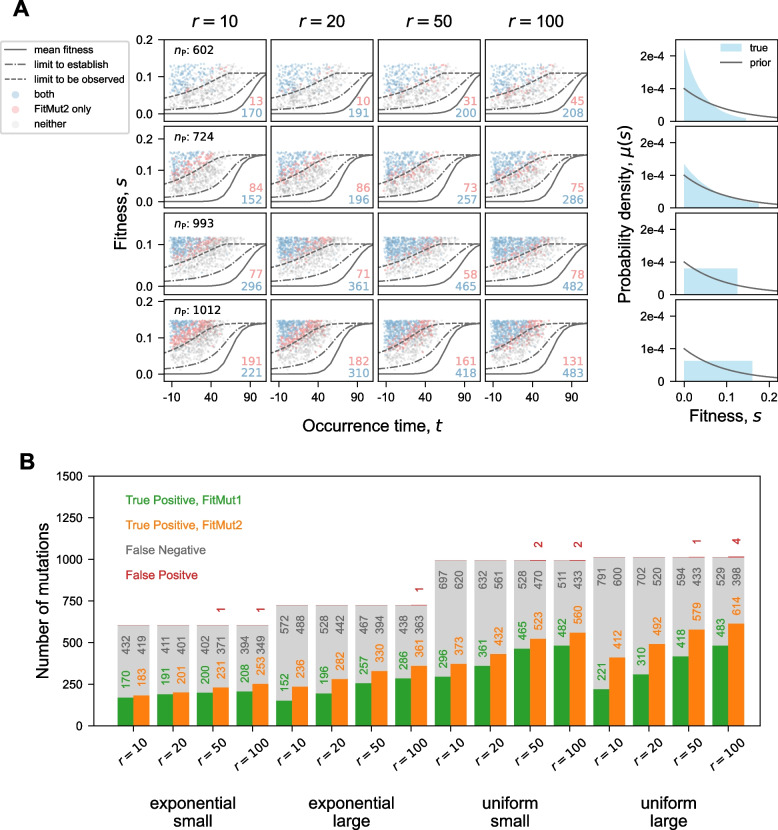
Detection ability for identifying adaptive mutations. **A** Each panel in the $4\times 4$ array corresponds to one simulation (Methods section). Each point represents an adaptive mutation that occurred and established in the simulation. Points are colored according to whether they were identified by both methods (blue), only by FitMut2 (pink), or by neither (grey) (no point that only by FitMut1); their counts are shown in the right bottom corner of each panel. $n_P$ represents the total number of established mutations for a given DFE. The three lines indicate the mean fitness (solid, $s = \bar{s}(t)$), the boundary above which mutations must occur in order to establish (dot-dashed, $s = \bar{s}(t+\frac{1}{s})$) and the boundary to be observed (short-dashed, $s = \bar{s}(t+\frac{1}{s} + \frac{1}{s}\ln \left( \frac{s \bar{n}_0}{c}\right) )$). The 5th column shows $\mu (s)$ and the prior $\tilde{\mu }(s)$ for each row. **B** Direct comparison of the detection ability between both algorithms

We examined how accurately FitMut2 estimates fitness effects and establishment times by comparing its inferences to the truth from our simulated dataset (Fig. 3). While numerous adaptive mutations are not detected by either algorithm, FitMut2 identifies hundreds of adaptive mutations missed by FitMut1 at low read number $r=10$ and a wide DFE (Results section), while maintaining a negligible false positive rate (Fig. 5B). For adaptive mutations detected by each algorithm, we compare inferred values of parameters to the truth in the simulation. For the adaptive mutations detected by FitMut2, there is a very strong correlation between the true fitness effect and the inferred value, and fairly strong correlation between the true occurrence times and the inferred establishment times. For comparison, we also show the results from FitMut1 in Fig. S2, and we see that our new algorithm significantly outperforms the old algorithm. To further assess inference accuracy for mutations identified as adaptive by both FitMut2 and FitMut1, we compared the estimation error between FitMut2 and FitMut1 (Fig. 4). FitMut2 has improved accuracy over FitMut1, particularly for those simulations in which FitMut1 could not estimate the mean fitness accurately. This improved accuracy is manifested in the error for FitMut2 typically having smaller magnitude than for FitMut1, even when they both make errors. For the simulations in which FitMut1 underestimates mean fitness, the fitness effects and establishment times are also underestimated, which affects the subsequent estimation of the DFE (see the 5th column in Figs. 3A and S2 A). When read depth is high or the variance of the DFE is smaller, the two methods produce similar results, though still with FitMut2 on average producing estimates closer to the ground truth.

We also compared the number of adaptive mutations missed by each algorithm, as well as the number of false positives (Fig. 5B). It should be emphasized that the vast majority of false negatives fall below the observation limit as detailed below, and are not expected to be detected. Additionally our algorithm reports an estimate of the uncertainty in our inferred values of *s* and $\tau$, which is based on the Hessian of the posterior likelihood at the optimal values of *s* and $\tau$ (details in Supplement S6).

### FitMut2 identifies mutations closer to the limit of detection

The clonal interference regime imposes a limit on the fitness effects that can establish, as well as those that can be detected. Although mutations occur throughout the experiment, only those that rise to a large enough frequency can be detected. Following [18], we restate the rough requirements for establishment and detection of a beneficial mutant. For an adaptive mutation with fitness effect *s* in a birth-death process with individual offspring-number variance per generation 2*c*, it takes $\sim \frac{1}{s}$ generations for the mutation to establish, and another $\sim \frac{1}{s}\ln \left( \frac{s \bar{n}_0}{c}\right)$ generations for the established mutation to sweep through an appreciable fraction of the lineage to be detectable. Here, $\bar{n}_0$ is the average effective lineage size, which in our case is 100. Therefore, typically, a mutation should satisfy $s\ge \bar{s}(t+\frac{1}{s})$ to establish, and also satisfy $s \ge \bar{s}(t + \frac{1}{s} + \frac{1}{s}\ln \left( \frac{s \bar{n}_0}{c}\right) )$ to be observed. Paired values *s* and *t* that satisfy these equations are found numerically and shown in Fig. 5A. We see that in Fig. 5A, as expected, identified adaptive mutations lie largely above the highest line.

FitMut2 is better able to identify mutations closer to the bound of detectability, as evidenced by the predominance of red points just above the uppermost curve in Fig. 5A. However, it has a nonzero false positive rate when the read depth is large (Fig. 5B). One way to combat the identification of false positives is to increase the threshold for designating a mutation as adaptive. In this work, the threshold is 1/2: we therefore deem a lineage adaptive if our Bayesian estimate says that the probability of it being adaptive is greater than 1/2. Increasing this threshold should lower our false positive rate.

## Discussion

In this work we have extended a previously-devised algorithm to infer mutation effects and establishment times from lineage trajectories over time. Using simulated data we have shown that our new algorithm FitMut2 performs better than the previous version FitMut1 when the read depth is low or the distribution of fitness effects is broad. By inferring the population mean fitness and single mutation effects self-consistently, instead of relying on the decline of neutral lineages, we can apply our algorithm to datasets with shallower sequencing, rapidly adapting populations, or smaller initial lineage sizes. However there are a number of aspects of this algorithm that deserve additional comments.

### Branching process model

The model of a growing lineage (details in Supplement S10) that we use to derive the distribution of read number conditional on a past measurement assumes that the lineage is reproducing and dying at constant rates in time, and that the difference between these rates constitutes the fitness. However, in the serial batch culture experiment (and in simulation), the population grows by two orders of magnitude ($\sim 2^8$) with minimal death every batch culture cycle — and this changes theoretical expectations for the distribution of offspring number from one measurement to the next. This can mostly be absorbed into an effective population size which is *g* times the bottleneck size (details in Supplement S3). However for large-effect mutations, the growth stochasticity during a single cycle may obey different statistics and a more careful analysis of the effective parameters is needed.

### Independence of sequencing noise across time points

One shortcoming of our approach (and that of FitMut1) is the assumption that the distribution of read number for a given lineage at a time point $t_k$ depends only on the number of reads counted at the previous time point: this approximation makes the problem of maximizing likelihood much more tractable. However, sequencing noise at different time points is uncorrelated: therefore if sequencing noise caused the read number to be large at the previous time point, there is no reason to believe that the subsequent measured read number would have a larger mean. The independence of sequencing noise across time points could be used to our advantage, allowing us to separate the stochasticity from cell division (which is of biological interest) from that due to sequencing noise. Though we have not pursued this direction in the current work, it remains a promising avenue. Previous work [18] has measured the value of *c* through doing multiple sequencing replicates — but the presence of multiple time points could allow us to circumvent the need for this extra sequencing.

### Choice of prior

To infer the fitness effect and establishment time of a mutation, we must choose a prior distribution $\tilde{\mu }(s)$ on which to run our Bayesian inference. In this work we consistently used an exponential prior $\tilde{\mu }(s) = U_b \lambda ^{-1}e^{-s/\lambda }$ with $\lambda = 0.1$ and $U_b=10^{-5}$. The prior for *s* and $\tau$ was then $p(s, \tau ) = n_{i, 0} \tilde{\mu }(s) s/c$ with *s*/*c* factoring in the establishment probability and $n_{i, 0}$ accounting for effective size of lineage *i* at $t_0$. The prior does not depend on $\tau$, whose prior distribution we took to be uniform between $-100$ and 112 generations measured relative to the start of the experiment.

The use of an exponential prior in *s* assumes that there are no very large mutations — because if there were, we would be increasingly unlikely to recognize them. Therefore in situations where the distribution of fitness effects is broader, an exponential prior may fail to identify many adaptive mutations, and a uniform prior may be more appropriate. The effect of the prior is further discussed in Supplement S7, where we conclude that our choice of prior makes little difference for the inferred $s_i$ in the majority of adaptive lineages. However, other aspects of our inference algorithm would also lose accuracy for large effect mutations, as discussed in Supplement S2.

To lessen the arbitrariness of our choice for $\tilde{\mu }(s)$, we could consider having a dynamically updated prior that starts as uniform and is updated based on the mutations identified as adaptive over successive iterations of our algorithm. It is conceivable that this would further increase our detection power. But there would be issues of low resolution in this empirically determined prior distribution, particularly for early iterations. Future work is needed to investigate how to iteratively update the prior distribution, and whether this further improves estimation accuracy.

### Rebarcoding and measurements of epistasis

As mentioned previously, our simulations allowed a maximum of one beneficial mutation per individual, since the DFE for a second mutation might be substantially different from that of the first [22]. The effects of recurrent beneficial mutations can be studied systematically using genetic re-barcoding of lineages [24], where a similar fitness-estimation algorithm has been implemented by iteratively inferring mean fitness and individual fitness effects. However in our work we infer establishment times of mutations from their lineage trajectories rather than from phylogeny information observable from the rebarcoding process as in Ref. [24].

### Computational performance

The most computationally expensive step in both FitMut2 and FitMut1 is the evaluation of the probability of being adaptive for each lineage, and the subsequent maximization of the posterior likelihood for those deemed adaptive. However, this step can readily be parallellized (in both FitMut2 and FitMut1) since each lineage may be handled independently. This substantially speeds up our algorithm, and we have included an option to parallellize computation using the python package multiprocess, which distributes iterations of the longest for loop in the program over multiple CPUs if available. With parallelization enabled, on our simulated dataset of $4\times 10^4$ lineages sampled over 15 time points, FitMut2 took around 1 minute per iteration on a laptop with 8GB of memory. In comparison, FitMut1 took around 15 minutes in total.

## Supplementary Information

**Additional file 1.**

## Data Availability

All of our code for simulations and inference, as well as the code to generate the figures in this paper, is available on GitHub at https://github.com/FangfeiLi05/FitMut2.
