# Supplementary material for: An improved algorithm for inferring mutational parameters from bar-seq evolution experiments
Source: BMC Genomics. 2023 May 6;24:246. doi: 10.1186/s12864-023-09345-x (PMC10164349; doi:10.1186/s12864-023-09345-x)
Supplement: Supplementary file 1 — Additional file 1. [file 12864_2023_9345_MOESM1_ESM.pdf]

Supplementary Information

S1. Notation

Symbol	Definition
T	total number of generations in evolution experiment
L	number of barcoded lineages
g	number of generations between successive cell transfers
$t_0, t_1, t_2, \ldots, t_K$	list of sequencing timepoints ($K+1$ in total)
$r_{i,k}$	read number of lineage i at t_k
R_k	total sequencing read depth at t_k, given by $R_k = \sum_{i=1}^{L} r_{i,k}$
$f_{i,k}$	frequency of lineage i at t_k, given by $f_{i,k} = r_{i,k}/R_k$
B_k	total number of cells transferred at bottleneck occurring at t_k
N_k	effective population size at t_k, given by $N_k = gB_k$
$n_{i,k}$	effective size of lineage i at t_k, given by $n_{i,k} = f_{i,k}N_k$
s_i	fitness effect of a mutation arising in lineage i
τ_i	establishment time of a mutation in lineage i
$\bar{s}(t)$	mean fitness of the population at time t
$\mu(s)$	distribution of fitness effects (DFE) of mutations
U_b	total beneficial mutation rate, given by $U_b = \int_0^\infty \mu(s)ds$
$2c$	offspring number variance perindividual per generation<br>(in branching process model)
$2\kappa_k$	phenomenological parameter capturing per-read variance in<br>offspring number between t_{k-1} and t_k
$\mathcal{E}_u^v$	reduction in frequency due to mean fitness, defined as<br>$\mathcal{E}_u^v = \exp\left[-\int_u^v \bar{s}(t')dt'\right]$
Θ_i	indicator variable for the establishment of a mutation occurring in lineage i

Table S1: Definitions of commonly used quantities.

S2. Definition of fitness

Evolutionary theory commonly uses two different definitions of fitness: *Malthusian* fitness and *Wrightian* fitness. Let t denote the elapsed time (measured in generations), and $n(t)$ denote the number of cells at time t. Malthusian fitness s_m is defined as the exponential growth rate (per generation) of the population, and Wrightian fitness s_w is defined as the number of offspring of a cell per generation. So for deterministic growth without death, these definitions imply

$$n(t) = n(0)e^{s_m t}, \tag{S1}$$

$$n(t) = n(0)\left(1 + s_w\right)^t. \tag{S2}$$

The fitness of a mutation in our simulations is defined in the Wrightian sense, which is natural for discrete generations. The branching process in the theory for our inference algorithm uses fitness in the Malthusian sense, which arises naturally in continuous time. Malthusian and Wrightian fitness are related by $s_m = \ln(1 + s_w)$ and are approximately equal when the fitness effect per generation is small, as is the case in our analyses. For fair comparison, we convert the mean fitness and fitness effect in the simulation from Wrightian fitness to Malthusian fitness when comparing the ground truth of the simulation to the inferred value.

In a well-mixed environment without ecological interactions, all cells in the population compete against the mean fitness. For an adaptive lineage i that originated from a single mutant cell, when the fitness of lineage, s_i, is larger than the mean fitness, the lineage increases in frequency. Once the mean fitness passes the fitness of the lineage, the lineage begins to decrease in frequency. Over time, the mean fitness increases as fitter adaptive lineages become enriched. If the total population size is fixed and we track lineage frequencies $\{f_{i,k}\}$ at each timepoint subject to $\sum_i f_{i,k} = 1$, one can see that they obey the equation

$$f_{i,k+1} \approx f_{i,k} e^{s_i(t_{k+1}-t_k)} \mathcal{E}_{t_k}^{t_{k+1}}, \tag{S3}$$

where $\mathcal{E}_{t_k}^{t_{k+1}}$ is defined in Table S1 and $\bar{s}(t) = \sum_i s_i f_i(t)$, which varies continuously in time. Note that this definition of $\bar{s}$ relies on the product of the individual fitnesses $\{s_i\}$ and the time between samples being small. When this is not true, it is still possible to jointly infer the individual lineage fitnesses without making use of the mean fitness (see Ref. [3]), but we have not pursued this here, in favor of explicitly accounting for the fraction of each lineage that carries a beneficial mutation (Section S5). This choice is likely to decrease the validity of our inference algorithm when mutations of large fitness effect arise.

Equation S3 is used to estimate the average read number of a lineage conditioned on its read number at the previous timepoint — however since we only infer the mean fitness at timepoints that are sequenced, we interpolate $\bar{s}$ linearly between these points of reference in order to calculate $\mathcal{E}$. Under this approximation,

$$\mathcal{E}_{t_k}^{t_{k+1}} \approx \exp\left[-\frac{(t_{k+1} - t_k)\left(\bar{s}(t_k) + \bar{s}(t_{k+1})\right)}{2}\right]. \tag{S4}$$

S3. Effective process

As discussed in the supplementary information (SI) of Ref. [1] and in Section S10, the process of population growth for g generations followed by $1:2^g$ dilution can be described as a time-homogeneous branching process if one chooses the correct effective values of $n(t)$ (effective lineage size) and $2c$ (offspring number variance per-individual). Specifically, for lineage i, the process of *a growth cycle* (growth of g generations followed by $1:2^g$ dilution) from t_{k-1} to t_k can be described as a single step of a branching process with effective lineage size $n_{i,k}$ given by g times the number of cells transferred at the dilution, and $2c$ given by the per-individual variance per growth cycle from the batch culture. Equivalently, one could define the effective size of each lineage as its actual size at bottleneck, and $2c$ reduced from the variance per cycle by a factor of g. In this work, we use the former convention.

In our simulated model with stochastic cell doubling and dilution noise, the per-individual variance in offspring number per growth cycle can be calculated using the law of total variance, and is $2c \approx 2$, with g generations of stochastic cell doubling introducing a variance of ≈ 1 (for $g \gg 1$), and $1:2^g$ dilution introducing another variance of 1. Therefore, the value of c which describes the simulated process in terms of the branching process model is $c = 1$. However, in real data the value of c is unknown, and is in fact of biological interest. Inferring it from our data is a future goal (as mentioned in the Discussion of the main text). In the current framework, when we infer our fitnesses, setting $c = 1$ only affects the establishment size of a beneficial mutation during the inference process, and we therefore expect that it does not much change our results.

S4. Parameterizing noise

Barcoded evolution experiments involve two processes: 1) a series of batch cultures, during which in each cycle the population grows for about g generations and then is diluted by a factor of 2^g into the next batch, and 2) sampling and preparation of cells via genomic DNA extraction, PCR and sequencing. As discussed in the SI of [1], both processes introduce stochasticity in the number of reads per barcode at each timepoint. Dealing with this stochasticity is essential for detecting adaptive mutations and for estimating their mutational parameters.

As we have discussed, the first step (cell growth and transfer) can be modeled as the branching process (Section S3). The second step (DNA extraction, PCR and sequencing) is also effectively a branching process, now composed with the results of the first step. The stochasticity coming from these processes is essentially a consequence of the compounding of Poisson counting statistics over time, often referred to as birth-death stochasticity, and means that the variance of $r_{i,k}$, $\mathrm{Var}(r_{i,k})$, is proportional to its expected value, $\langle r_{i,k}\rangle$. The constant of proportionality is given by a phenomenological parameter $2\kappa_k$, which captures all sources of noise (cell growth and dilution, DNA extraction, PCR and sequencing). Under this noise model, $\mathrm{Var}(r_{i,k}) = 2\kappa_k\langle r_{i,k}\rangle$ for every lineage i. In order to determine the values of the κ_k for each timepoint t_k, we first find all lineages with read number at t_{k-1} being $r_{i,k-1} = q$. Then we estimate the mean μ and the variance σ^2 of $r_{i,k}$ conditioned on $r_{i,k-1} = q$. Then, conditional on q, $\kappa_k = \sigma^2/(2\mu)$. We average κ_k over all $q \in [20, 30]$, which are likely to correspond to neutral lineages for typical read depths (see also the SI of Ref. [1]). Although we only determine the values of κ_k once and for all timepoints at the beginning of our inference algorithm, we have found that our results are quite insensitive to the values of the $\{\kappa_k\}$ inferred.

S5. Algorithm details

Here we describe in detail the algorithm executed by `FitMut2`, which is similar to the algorithm implemented in Ref. [3], the main difference being the inference of establishment times from the lineage trajectories rather than from phylogenetic relationships observable from repeated barcoding. The basic premise is Bayes' theorem: we wish to know the probability that each lineage is adaptive, conditional on our observed lineage trajectory $\{r_{i,k}\}$. In this and the subsequent section, we assume that the lineage index is always i, and thus suppress the i subscript. Bayes' theorem tells us that for a lineage with observed lineage trajectory $\{r_k\}$,

$$P(\Theta = \theta|\{r_k\}) = \frac{p(\{r_k\}|\Theta = \theta)P(\Theta = \theta)}{p(\{r_k\})}, \tag{S5}$$

where Θ is an indicator variable that is 1 if the lineage contains an established mutation and 0 otherwise.

When $\theta = 1$, we can further decompose this adaptive hypothesis into an integral over all possible ways to be adaptive, with a prior distribution $p(s, \tau)$ over s and τ. Thus,

$$p(\{r_k\}|\Theta = 1)P(\Theta = 1) = \int p(\{r_k\}|s, \tau)p(s, \tau)dsd\tau. \tag{S6}$$

When $\theta = 0$ we use the prior $P(\Theta = 0) \approx 1$, since the vast majority of lineages do not obtain a beneficial mutation (or equivalently $\int p(s, \tau)dsd\tau \ll 1$). Therefore, we reduce the problem of inference to calculating: 1) the probability of the $\{r_k\}$ given the neutral hypotheses and 2) the probability of the $\{r_k\}$ given the adaptive hypothesis with a mutation having fitness effect s and establishment time τ. Together with a prior distribution over s and τ, this gives us the tools to assess the probability that a lineage is adaptive.

Note that we have split the hypothesis into a neutral and adaptive case, instead of inferring an s and τ for each lineage, for two reasons: The first is that this split allows us to save computation time, by only finding the most likely s and τ for those lineages deemed likely enough to be adaptive (which is a small fraction of all lineages). The second is that if we were to have a single prior distribution for s, which had support at 0, then some lineages might be assigned very small fitness effects, which may not reflect real beneficial mutations.

We now explain how to calculate the various terms needed to evaluate the likelihood of a lineage trajectory under the two different scenarios.

S5.1. Neutral hypothesis

To calculate $p(\{r_k\}|\Theta = 0)$, we make an important approximation. First we note that

$$p(\{n_k\}|\Theta = 0) = p(n_0|\Theta = 0)\prod_{k=1}^{K} p(n_k|n_{k-1}, \Theta = 0). \tag{S7}$$

In other words, the *effective cell number* at each timepoint obeys a Markov process, and the distribution of n_k only depends on the value of n_{k-1}. Note that strictly speaking, the analogous statement for the read numbers $\{r_k\}$ is false: The distribution of r_k does not only depend on r_{k-1}, due to independent sequencing noise introduced

at each timepoint. Strictly, for the read numbers $\{r_k\}$,

$$p(\{r_k\}|\Theta=0) = \int p(\{n_k\}|\Theta=0)p(\{r_k\}|\{n_k\}) \prod_{k=0}^{K} dn_k$$
$$= \int p(n_0|\Theta=0) \prod_{k=1}^{K} p(n_k|n_{k-1},\Theta=0) \prod_{k=0}^{K} p(r_k|n_k) \prod_{k=0}^{K} dn_k. \quad \text{(S8)}$$

Nonetheless, we make the approximation that the joint distribution of the $\{r_k\}$ factorizes in the same way as that of the $\{n_k\}$. Though we expect that this assumption become less accurate at low read depth, where the independent noise at each timepoint is larger, empirically our algorithm still seems to be accurate over the range of parameters we have tested. We additionally investigated an alternative method that does not make this assumption, and instead tries to jointly infer the sequence of cell numbers $\{n_k\}$ that is most likely to have produced our data. However, we have not pursued this method in the current work, due to its infeasibility under the adaptive hypothesis.

With the assumption of Markovian dynamics for the $\{r_k\}$, we define the likelihood of our data given the neutral hypothesis as

$$p(\{r_k\}|\Theta=0) = p(r_0|\Theta=0) \prod_{k=1}^{K} p(r_k|r_{k-1},\Theta=0), \quad \text{(S9)}$$

where for $1 \leq k \leq K$, according to Equation S30,

$$p(r_k|r_{k-1},\Theta=0)$$
$$=\frac{1}{\kappa_k}\sqrt{\frac{\langle r_k|r_{k-1}\rangle}{r_k}} \exp\left[-\frac{r_k+\langle r_k|r_{k-1}\rangle}{\kappa_k}\right] I_1\left(\frac{2\sqrt{\langle r_k|r_{k-1}\rangle r_k}}{\kappa_k}\right). \quad \text{(S10)}$$

Here I_1 is the modified Bessel function of the first kind, and the notation $\langle r_k|r_{k-1}\rangle$ indicates the expected value of r_k conditional on the value of r_{k-1} and $\Theta = 0$. This value is given by the expression for the decline of a neutral lineage in the presence of an increasing mean fitness,

$$\langle r_k|r_{k-1}\rangle = r_{k-1}\mathcal{E}_{t_{k-1}}^{t_k} \frac{R_k N_{k-1}}{R_{k-1}N_k}. \quad \text{(S11)}$$

We can then take the product of these conditional distributions as specified in Equation S9, in order to get the likelihood of our data under the neutral hypothesis. However we must deal separately with the first timepoint which gives us a factor of $p(r_0)$. This is discussed below.

S5.2. Adaptive hypothesis

For the adaptive hypothesis, we have

$$p(\{r_k\}|s,\tau) = p(r_0|s,\tau) \prod_{k=1}^{K} p(r_k|r_{k-1},s,\tau). \quad \text{(S12)}$$

When an adaptive mutation occurs and establishes in a lineage, the lineage can contain both mutant and neutral cells which are indistinguishable on the basis of barcode alone. Before the establishment, the neutral cells

comprise almost the entire lineage, while after an adaptive mutation establishes, the mutant cells begin to sweep through the lineage.

In order to calculate the likelihood of our data under the adaptive hypothesis, we use Equation S10, but condition on a value of s and τ rather than $\Theta = 0$, and with the conditional expectations $\langle r_k | r_{k-1} \rangle$ calculated under the assumption of a given s and τ (Equation S13). Assuming that an adaptive mutation with fitness effect s establishes at time τ in a lineage, the average read number of a lineage at timepoint t_k is

$$\begin{aligned} \langle r_k | r_{k-1} \rangle &= \langle n_k \rangle R_k / N_k, \text{ with} \\ \langle n_k \rangle &= \left(r_{k-1} N_{k-1} / R_{k-1} - n_{k-1}^{\text{mut}} + n_{k-1}^{\text{mut}} e^{s(t_k - t_{k-1})} \right) \mathcal{E}_{t_{k-1}}^{t_k}, \text{ and} \\ n_k^{\text{mut}} &= \frac{c}{s - \bar{s}(\tau)} \mathcal{E}_{\tau}^{t_k} \exp s(t_k - \tau) \mathbf{1}_{\{t > \tau\}}. \end{aligned} \tag{S13}$$

Here, $\mathbf{1}_{\{t>\tau\}}$ is an indicator variable that is 1 if $t > \tau$ and 0 otherwise, and we have used the fact that the establishment size of a lineage beyond which it grows deterministically (as if it had started at this size at its establishment time) is given by $\frac{c}{s - \bar{s}(\tau)}$.

We assume that $n_{k-1}^{\text{mut}} = r_{k-1} N_{k-1} / R_{k-1}$ if $r_{k-1} N_{k-1} / R_{k-1} < n_{k-1}^{\text{mut}}$. Furthermore, we impose a lower cutoff on $s - \bar{s}(\tau)$ to avoid very large establishment sizes.

Since we also want to calculate likelihoods for $\tau < 0$ (corresponding to mutations that occurred during the pre-growth phase), we need the mean fitness before $t = 0$ when calculating $\mathcal{E}_{\tau}^{t_k}$: We assume that the mean fitness is 0 before the experimental evolution begins.

As in the case of the neutral hypothesis, we also need to deal with the first sequencing timepoint t_0, for which we do not have a previous timepoint on which to condition. Therefore for the first timepoint we define

$$p(r_0 | \Theta = 0) = p(r_0 | s, \tau) = p(r_0), \tag{S14}$$

with

$$p(r_0) = \frac{1}{\kappa_0} \sqrt{\frac{\langle r_0 \rangle}{r_0}} \exp\left(-\frac{r_0 + \langle r_0 \rangle}{\kappa_0} \right) I_1 \left(\frac{2\sqrt{\langle r_0 \rangle r_0}}{\kappa_0} \right). \tag{S15}$$

Here, $\langle r_0 \rangle = r_0$. In other words, the mean of r_0 is approximated by the measured value of r_0 for each lineage. κ_0 is assumed to be 2.5, which is empirically a typical value. Estimating the most likely value of n_0 and the resulting distribution of r_0 from the data is an interesting problem which we leave for future work.

Therefore, under the adaptive hypothesis, the probability of observing our data is given by

$$p(\{r_k\} | \Theta = 1) P(\Theta = 1) = \int F(s, \tau) ds d\tau. \tag{S16}$$

where we define the *posterior likelihood* $F(s, \tau)$ as

$$F(s, \tau) = p(s, \tau) p(r_0) \prod_{k=1}^{K} p(r_k | r_{k-1}, s, \tau), \tag{S17}$$

with prior distribution

$$p(s, t) = \tilde{\mu}(s) \frac{s}{c} \frac{\langle r_0 \rangle N_0}{R_0}, \quad \tilde{\mu}(s) = \frac{U_b}{\lambda} \exp\left[-\frac{s}{\lambda} \right], \quad U_b = 10^{-5}, \quad \lambda = 0.1. \tag{S18}$$

Therefore, to calculate the probability that a lineage is adaptive, we numerically integrate $F(s, \tau)$ for that lineage over s and τ. Since this calculation for each lineage is independent, it can be readily parallelized (see Discussion in the main text).

Since $P(\{r_k\}) = P(\{r_k\}|\Theta = 0)P(\Theta = 0) + P(\{r_k\}|\Theta = 1)P(\Theta = 1)$, we can then use Equation S5 to calculate the *probability* that our lineage is adaptive or neutral given the data, that is, $P(\Theta = 0|\{r_k\})$ and $P(\Theta = 1|\{r_k\})$. If $P(\Theta = 1|\{r_k\}) > 0.5$, we conclude that the lineage contains an established mutation — though this threshold can be changed depending on the use case. The fitness effect and establishment time that we infer for this lineage are given by the s and τ that maximize the logarithm of posterior likelihood, $\ln F(s, \tau)$. This maximization is discussed in the next section.

S5.3. Numerical optimization

The fitness effects and establishment times of adaptive mutations are inferred by optimizing the posterior likelihood (Section S5) over the s and τ. There are several choices for how to do this optimization. The most straightforward is to perform a direct search: evaluating $\ln F(s, \tau)$ on a grid of points and finding the s and τ which yield the maximum value. This is the default mode of operation of our algorithm, and works due to the low dimensionality of our search space. However we have also provided alternate gradient-free optimization routines, such as the Nelder Mead algorithm and the differential evolution algorithm. The results presented in this work use the Nelder Mead optimization algorithm.

Figure S5 shows the landscape of the log-posterior likelihood for an adaptive lineage in which an adaptive mutation established, as well as the landscape of the log-posterior likelihood for a neutral lineage. We see that $F_i(s, \tau)$ is not particularly sharply peaked in all directions — in fact its density is strongly concentrated along a one-dimensional curve in two dimensions. Though the Nelder mead algorithm accurately finds the maximum in the likelihood landscape, there is some mismatch between the location of this maximum and the true values of the fitness and occurrence times.

S5.4. Updating the mean fitness

Since the algorithm proceeds iteratively, we need a rule for updating the mean fitness after the fitnesses of individual lineages are inferred. Specifically, at the end of each iteration, we estimate the effective number of mutant cells for each lineage, $n_{i,k}^{\text{mut}}$, for each timepoint t_k, given the s_i and τ_i that were inferred. Then we update the mean fitness as

$$\bar{s}(t_k) = \frac{\sum_i n_{i,k}^{\text{mut}} s_i}{\sum_i n_{i,k}}. \tag{S19}$$

We can now proceed by re-inferring the individual lineage fitnesses given our updated mean fitness.

S6. Error estimation

In addition to estimating s and τ for each adaptive lineage, we provide uncertainties of our estimates, which can be calculated via the Hessian matrix of $\ln F$, evaluated at maximal point (s_0, τ_0) found by our optimization algorithm. The Hessian is given by

$$H(s_0, \tau_0) = \begin{pmatrix} \partial_{ss} \ln F & \partial_{s\tau} \ln F \\ \partial_{\tau s} \ln F & \partial_{\tau\tau} \ln F \end{pmatrix} \Bigg|_{s_0, \tau_0} . \tag{S20}$$

The eigenvalues of H give information about how sharp of a maximum in $\ln F$ we have found — if the maximum is very shallow we are more uncertain of its location, and therefore of our estimates of s and τ. Following Ref. [1], we calculate the eigenvectors $\vec{v}_1$, $\vec{v}_2$ and eigenvalues λ_1, λ_2 of H. By projecting the eigenvectors along the s and τ directions of our parameter space and using the fact that F is locally Gaussian with scale $1/\sqrt{\lambda_i}$ in the direction $\vec{v}_i$, we define the error in s to be $\max(\vec{v}_1 \cdot \tilde{s}/\sqrt{\lambda_1}, \vec{v}_2 \cdot \tilde{s}/\sqrt{\lambda_2})$, and the error in τ to be $\max(\vec{v}_1 \cdot \tilde{\tau}/\sqrt{\lambda_1}, \vec{v}_2 \cdot \tilde{\tau}/\sqrt{\lambda_2})$, where $\tilde{s} = (1, 0)$ and $\tilde{\tau} = (0, 1)$ are the unit vectors respectively.

S7. Effect of prior

The choice of prior $\tilde{\mu}(s)$ affects the landscape over which we optimize $\ln F(s,\tau)$. Ignoring logarithmic terms, which have a negligible effect on the location of the maximum of F, we can expand our objective function as

$$\ln F(s,\tau) \sim -\alpha(s-x)^2 + \beta + \ln \tilde{\mu}(s) \quad \text{with} \quad \alpha = \left| \frac{1}{2} \partial_{ss} \ln F(s,\tau)|_{s=x} \right| \tag{S21}$$

in the neighborhood of the optimum, where x is the optimal s that would be found under a uniform prior. Therefore if we use a prior $\tilde{\mu}(s) \sim e^{-s/\lambda}$, we find that the optimal s where F is extremized shifts by an amount $-\frac{1}{2\alpha\lambda}$. If the lineage does not contribute much to the mean fitness, this shift in the fitness of lineage does not affect the optimization landscape and the prior that we have chosen simply shifts $\hat{s}$ by an amount that depends inversely on α and λ.

From Figure S5 we estimate that $\alpha \approx 20/.05^2 = 8000$. Therefore varying λ from 0.1 (its value in this work) within an order of magnitude would have little effect on $\hat{s}$.

S8. DFE estimation

In this work, in addition to inferring the fitness effects of individual mutations, we have also inferred the distribution of fitness effects $\mu(s)$. Since the fitness effect of a mutation affects how likely it is to be seen, we need a way to get $\mu(s)$ from the observed fitness effects.

Let $f(s,t)$ be the total fraction of cells in the population with fitness effect in the range $[s, s+\delta s]$ at the time t. According to Ref. [1],

$$f(s,t) = \mu(s)\delta s \frac{c}{s} e^{st} + \mu(s)\delta s \ln(N_f \mu(s)\delta s) e^{st}, \tag{S22}$$

where the first term on the right side accounts for mutations during the evolution, and the second term accounts for mutations that arose during pre-growth. $N_f = 10^{12}$ is the maximum population size after barcoding, before the separation of the replicates.

Thus, the distribution of fitness effect (DFE), $\mu(s)$, satisfies the equation

$$\mu(s)\delta s = \frac{f(s,t) s e^{-st}}{c + s \ln\left(N_f \mu(s)\delta s\right)}. \tag{S23}$$

To solve $\mu(s)$ from equation S23, let

$$\begin{cases} a = \dfrac{c + s\ln(N_f)}{s}, \\ b = f(s,t)e^{-st}, \\ z = \ln\left(e^a \mu(s)\delta s\right). \end{cases} \tag{S24}$$

Equation S23 can be rewritten as equation $ze^z = be^a$ with solution $z = \text{ProductLog}\left(be^a\right)$. Thus, we have $\mu(s) = \frac{e^{z-a}}{\delta s}$.

Here, Equation S23 is an approximation that does not account for the mean fitness. Therefore, to infer $\mu(s)$ from our results, we choose the timepoint $t = 32$, at which time the mean fitness is typically still small.

S9. Simulation details

We have written simulations to produce data on which we have tested our algorithm, as a means to compare its results against a ground truth. The code for these simulations is included on GitHub along with our inference algorithm. In our simulations, during 16 generations of pre-growth, the number of offspring of a single cell with fitness s is distributed as $\text{Pois}(2(1+s))$. Here, $\text{Pois}(\lambda)$ represents Poisson distribution with parameter λ. This pre-growth allows lineage size to fluctuate, and introduces variability in the lineage size going into the pooled batch culture. At the end of the pre-growth phase, each cell i has grown into a colony of size n_i^*. To initialize the evolution experiment, an average of 100 cells per barcode are sampled: $n_i(0)$ cells from each lineage are sampled with

$$n_i(0) \sim \text{Pois}\left(\frac{100\hat{L}n_i^*}{\sum_i n_i^*}\right), \tag{S25}$$

where $\hat{L}$ is the number of non-extinct colonies. $\tilde{L}$ is then defined as the number of non-extinct lineages with $n_i(0) > 0$.

During first batch culture cycle, growth noise is simulated by updating the number of descendants of a single cell according to

$$n_i(t+1) \sim \text{Pois}\left(\frac{n_i(t)\,(1+s_i)}{\sum_i n_i(t)\,(1+s_i)} 100\tilde{L} \times 2^t\right). \tag{S26}$$

After g generations, the cells which get transferred to the next batch are sampled randomly from the final population and are Poisson distributed with mean $n_i(g)/2^g$.

Of the four DFEs in our simulation, two are truncated exponential distributions, that is, $\mu(s) \sim \exp\left[-s/0.045\right]$ with $s \in (0, 0.145)$, and $\mu(s) \sim \exp\left[-s/0.075\right]$ with $s \in [0, 0.175]$. The other two are uniform between on intervals $(0, 0.125)$ and $(0, 0.16)$ respectively. All DFEs have $\int_0^\infty \mu(s)ds = 10^{-5}$.

In order to determine whether a mutation has established or not in simulation, we compare its instantaneous effective cell number $n(t)$ to $2/(s - \bar{s}(t))$, and if it crosses this size we record it as established — this heuristic means that its probability of extinction would be about e^{-2} if $\bar{s}$ stopped increasing thereafter. When we infer the mutational identity of lineages from experimental data, the establishment size used in our inference algorithm is $c/(s - \bar{s})$. However, for real data we do not know *a priori* what the value of c is in experimental data, and our algorithm assumes that $c = 1$.

S10. Distribution of offspring from a single individual

The time-homogeneous birth-death process is a simple model to understand the growth of a lineage that is subject to both stochastic drift and systematic selection. Let $n(t)$ denote a random variable that represents the number of individuals at the time t (where time is now taken to be continuous and in units of generations). Assume that each individual has a birth rate k_b and death rate k_d, and individuals grow or die independently of one another. In a small interval of time $\delta t \ll 1/k_b, 1/k_d$, each individual divides with probability $k_b \delta t$, dies with probability $k_d \delta t$, and does nothing with probability $1-(k_b+k_d)\delta t$. Let B_j and D_j respectively denote Bernoulli random variables indicating birth or death in the interval $[t, t+\delta t]$ for the jth individual. As done in Ref. [1], we define a moment generating function of this birth-death process

$$M(\phi, t) = \left\langle e^{-\phi n(t)} \right\rangle_{n(t)}, \tag{S27}$$

with $\langle X \rangle_Y$ denoting an average of the random variable X over the random variable Y. Since our branching process obeys the equation $n(t+\delta t) = \sum_{j=1}^{n(t)} [1 - D_j + B_j]$, we can derive a partial differential equation for this moment generating function

$$\frac{\partial M}{\partial t} = (s\phi - c\phi^2)\frac{\partial M}{\partial \phi}, \tag{S28}$$

where $s = k_b - k_d$ and $c = (k_b + k_d)/2$. We can then solve this equation by the method of characteristics to obtain

$$M(\phi) = \exp\left[\frac{-n(0)e^{st}\phi}{1+\frac{c}{s}(e^{st}-1)\phi}\right]. \tag{S29}$$

We finally get the probability distribution $p(n)$ of the cell number n at time t, starting from $n(0)$ at time 0, which is given by the inverse Laplace transform of $M(\phi)$,

$$p(n) = \frac{1}{b}\sqrt{\frac{a}{n}}\exp\left(-\frac{n+a}{b}\right) I_1\left(\frac{2\sqrt{an}}{b}\right), \tag{S30}$$

where $a = n(0)e^{st}$ and $b = \frac{c}{s}(e^{st}-1)$. Here, $I_1(x)$ denotes the modified Bessel function of the first kind ($I_1(x) = \sum_{k=0}^{\infty} \frac{1}{k!\,\Gamma(k+2)} \left(\frac{x}{2}\right)^{2k+1}$). The mean of this distribution is a and its variance is $2ab$. For n large compared to b^2/a, our distribution takes the limiting form

$$p(n) \approx \sqrt{\frac{a^{1/2}}{4\pi b n^{3/2}}}\exp\left[-\frac{(\sqrt{n}-\sqrt{a})^2}{b}\right]. \tag{S31}$$

Equation S31 is the theoretical distribution that `FitMut1` used for the number of reads at the current timepoint t_k conditioned on the previous timepoint t_{k-1}, where a is the mean number of reads at the current timepoint, and b is replaced by the κ_k. In `FitMut2`, we use Equation S30, which is more accurate for small read numbers. Equations S11 and S13 are used to calculate the expected number of reads corresponding to a particular barcode at the current timepoint, conditional on the read number at the previous timepoint, under neutral and adaptive hypotheses.

Our branching process can also tell us what the establishment size of a lineage is, above which it is destined to fix. Note that $P(n(t) = 0)$ is the extinction probability by time t. We can calculate this probability directly from the moment generating function according to

$$P(n(t) = 0) = \lim_{\phi \to \infty} M(\phi, t) = \exp\left[-n(0)\frac{e^{st}}{\frac{c}{s}(e^{st} - 1)}\right] \approx \exp\left[-n(0)\frac{s}{c}\right], \tag{S32}$$

where the last approximation obtains when $t \gg 1/s$, which corresponds to eventual probability of fixation. Therefore, the lineage of a single mutant cell (carrying an adaptive mutation with fitness effect $s \ll c$) goes extinct with probability $\approx 1 - s/c$ and establishes with probability s/c. Furthermore, once a lineage reaches size c/s, it is unlikely to go extinct. If we define an establishment size c/s then the establishment time τ is defined through the relation $n(t) = \frac{c}{s}e^{s(t-\tau)}$ (with $n(0) = 1$ meaning that the mutation occurred at time 0), which yields a t-independent distribution for $\tau - t$ as $t \to \infty$ [2, 1]. $\tau - t$ is asymmetrically distributed around 0 with width scaling as $1/s$, and $\tau - t$ much more likely to be large and positive than large and negative on this scale. However the most likely value of $\tau - t$ is 0, which allows us to compare the occurrence time from simulation to establishment time from inference.

References

[1] S. F. Levy, and J. R. Blundell, S. Venkataram, D. A. Petrov, D. S. Fisher, G. Sherlock, Quantitative evolutionary dynamics using high-resolution lineage tracking. *Nature* **519**, 181–186 (2015).

[2] M. M. Desai, D. S. Fisher, Beneficial mutation selection balance and the effect of linkage on positive selection. *Genetics* **176**, 1759–1798 (2007).

[3] A. N. Nguyen Ba, I. Cvijović, J. I. Rojas Echenique, K. R. Lawrence, A. Rego-Costa, X. Liu, S. F. Levy, M. M. Desai, High-resolution lineage tracking reveals travelling wave of adaptation in laboratory yeast. *Nature* **575**, 494-–499 (2019).

S11. Figures

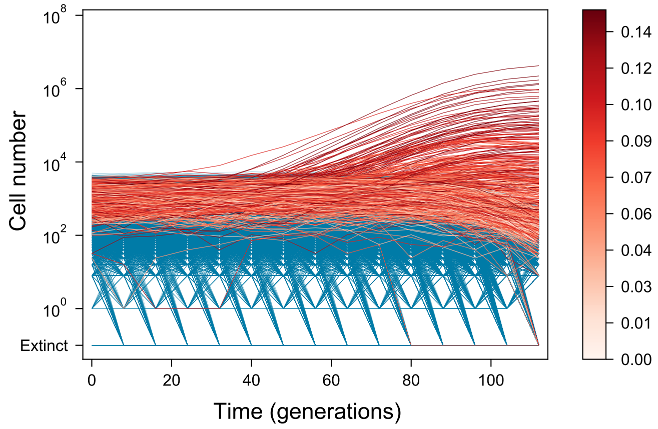

Figure S1: Trajectories of lineages from simulation. Lineages that contain an adaptive mutation that established are colored by the fitness effect of the adaptive mutation in the lineage. Neutral lineages are shown in blue. The simulation corresponds to the simulation in the 2th row, the 4th column in Figure S2

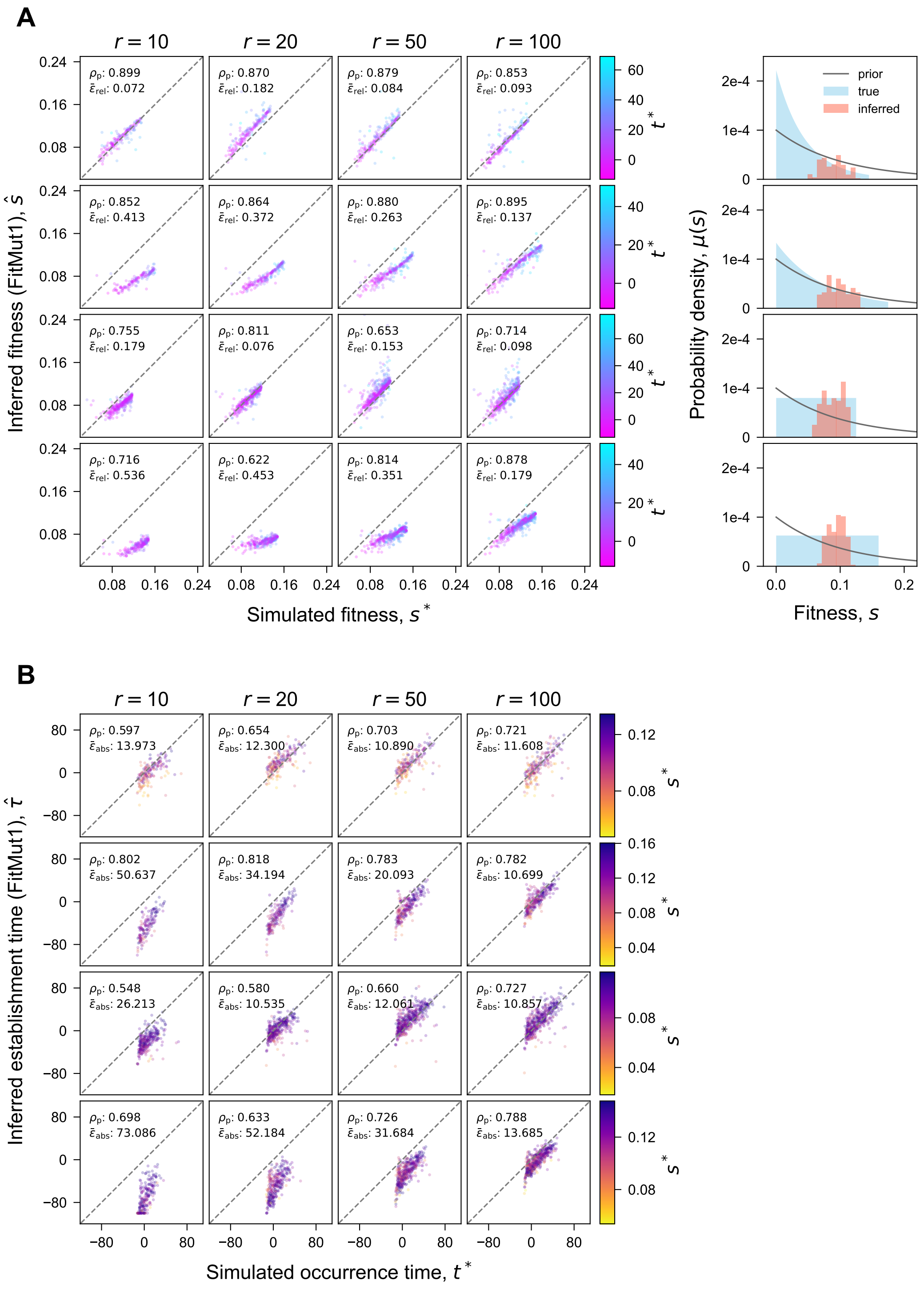

Figure S2: Inference accuracy of (A) fitness effects and (B) establishment times (`FitMut1`). Comparison of inferred s and τ with simulation. Compare this figure with equivalent Figure 3 for `FitMut2`. See caption of Figure 3 for parameter definitions. The inferred values are less accurate than the same results using `FitMut2`.

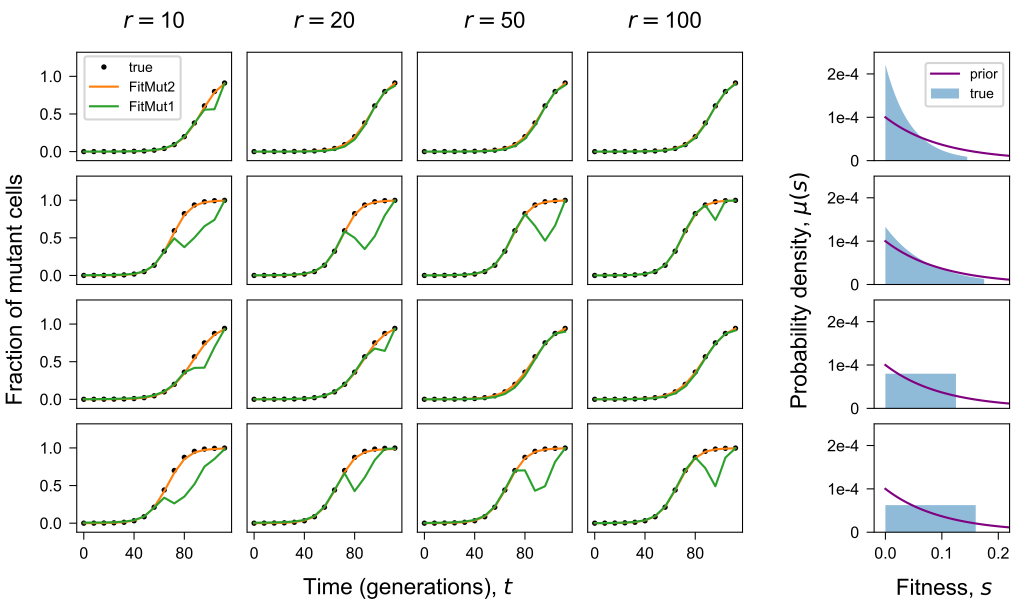

Figure S3: Mutant fraction of the population. Comparison of the estimated and true total frequency of mutant cells in the population over time. Each panel in the 4×4 array corresponds to one simulation. The 5th column shows the DFE $\mu(s)$ used in each simulation condition, and the prior $\tilde{\mu}(s)$ used for both `FitMut2` and `FitMut1`.

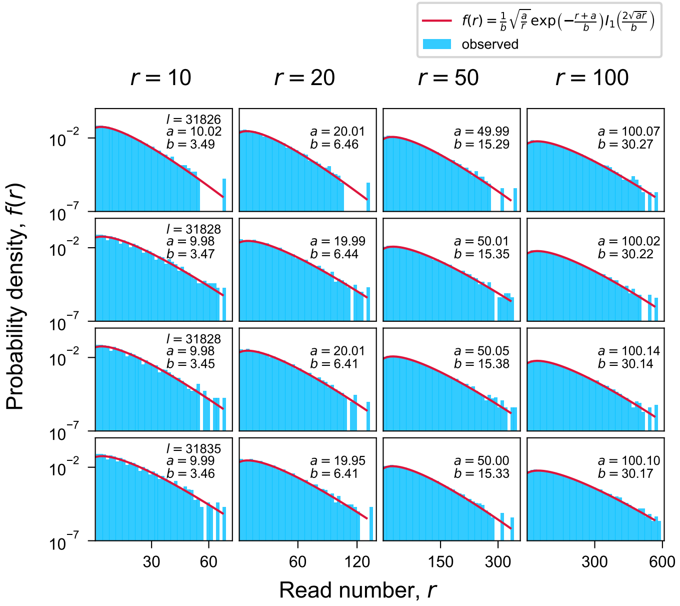

Figure S4: Probability distribution of read number. Probability distribution of the read number r at $t = 0$. Each panel in the 4×4 array corresponds to one simulation. The blue histogram is a plot of simulated data. The theoretical probability distribution (red line) is defined by $f(r) = \frac{1}{b}\sqrt{\frac{a}{r}}\,\exp\left(-\frac{r+a}{b}\right) I_1\left(\frac{2\sqrt{ar}}{b}\right)$, with a and $2ab$ being respectively the mean and variance of the simulated data. The theoretical read number distribution is fit to the observed distribution with parameters a and b. This indicates that our simulation of pre growth is well approximated by the critical branching process with no fitness differences between lineages.

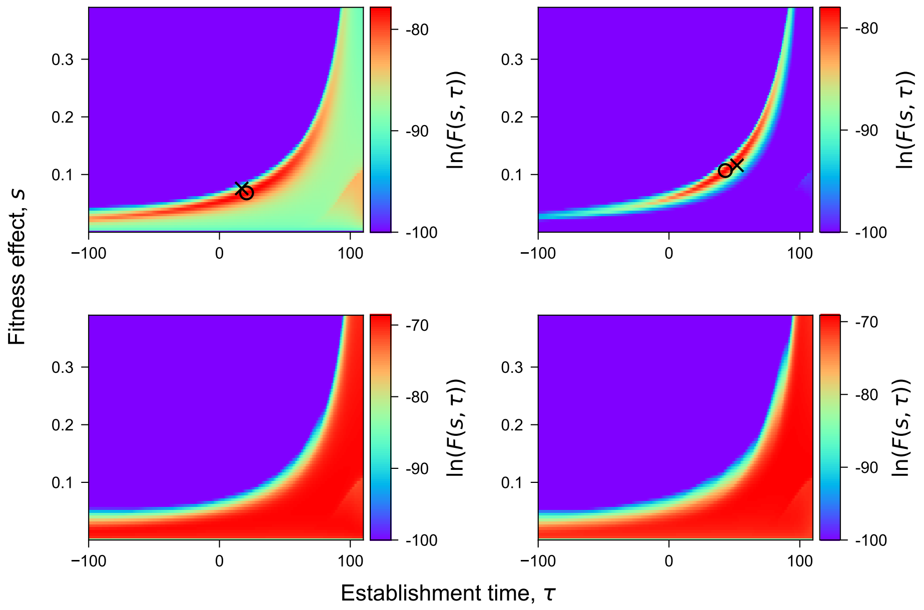

Figure S5: Optimization of the posterior likelihood. Value of the log-posterior likelihood $\ln F(s, \tau)$ for two adaptive lineages on the 1st row, and two neutral lineages on the 2nd row, in the simulation from the 1st row and 4th column of Figure 2. In the adaptive lineages, the fitness effect s and establishment time τ inferred by `FitMut2` is marked with $\circ$. The true fitness effect s^* and occurrence time t^* is marked with $\times$.
